# The protective effect of the intestinal microbiota in type-1 diabetes in NOD mice is limited to a time window in early life

**DOI:** 10.3389/fendo.2024.1425235

**Published:** 2024-09-26

**Authors:** Nerea Fernandez Trigo, Cristina Kalbermatter, Bahtiyar Yilmaz, Stephanie C. Ganal-Vonarburg

**Affiliations:** ^1^ Department of Visceral Surgery and Medicine, Inselspital, Bern University Hospital, Bern, Switzerland; ^2^ Department for BioMedical Research (DBMR), University of Bern, Bern, Switzerland

**Keywords:** type-1 diabetes, microbiota, window of opportunity, early life, weaning, autoimmunity, regulatory T cells

## Abstract

**Introduction:**

The incidence of type-1 diabetes is on the rise, particularly in developed nations, and predominantly affects the youth. While genetic predisposition plays a substantial role, environmental factors, including alterations in the gut microbiota, are increasingly recognized as significant contributors to the disease.

**Methods:**

In this study, we utilized germ-free non-obese diabetic mice to explore the effects of microbiota colonization during early life on type-1 diabetes susceptibility.

**Results:**

Our findings reveal that microbiota introduction at birth, rather than at weaning, significantly reduces the risk of type-1 diabetes, indicating a crucial window for microbiota-mediated modulation of immune responses. This protective effect was independent of alterations in intestinal barrier function but correlated with testosterone levels in male mice. Additionally, early life colonization modulated T cell subset frequencies, particularly T helper cells and regulatory T cells, in the intestine, potentially shaping type-1 diabetes predisposition.

**Discussion:**

Our findings underscore the pivotal role of early-life microbial interactions in immune regulation and the development of autoimmune diseases.

## Introduction

Type-1 diabetes (T1D) affects 8.4 million people world-wide and its incidence has been increasing in developed countries in the last decades (1–3). The disease is also prevalent in the younger population with 18% younger than 20 and 64% younger than 60 years of age (4). The median age of disease onset is 29 years of age, although peaks in younger children and adolescents have been previously reported (1, 4, 5). Genetic factors are linked to an elevated risk of developing T1D (6). Yet, only about 5% of individuals genetically predisposed to T1D actually manifest the disease (7), suggesting a predominant role for environmental influences. The specific molecular mechanisms underlying the observed increase in autoimmune and non-communicable diseases remain largely elusive. However, lifestyle changes and environmental shifts in Western societies are proposed to play contributory roles (8). Notably, alterations in the gut microbiota, characterized by diminished bacterial diversity and the loss of particular strains—phenomena encapsulated by the ‘disappearing microbiome hypothesis’—are increasingly recognized as significant drivers behind T1D pathogenesis (9–11).

The non-obese diabetic (NOD) mouse model is routinely used to study T1D *in vivo* (12). These mice are highly susceptible to the spontaneous development of diabetes, mediated by leukocyte infiltration and destruction of the pancreatic *β* cells. The onset of diabetes can be monitored by glycosuria and a non-fasting hyperglycemia. It has been demonstrated that the hygiene level of the animal husbandry positively correlates with the incidence of diabetes in NOD mice and that germ-free NOD mice are more prone to develop T1D than colonized control animals (13–15). The protective role of the presence of endogenous commensal microbiota for T1D development is, therefore, a common assumption. It is an increasingly believed notion that the right environmental cues during the perinatal and early life period, such as microbial signals, nutrition, and lifestyle in a larger sense, are game-changing for life-long immune regulation and health of the growing child (16, 17). Interestingly, early-life exposure to infectious agents confers greater protection from diabetes development than exposure later in life. For example, in NOD mice intravenous injections of the live bacille Calmette-Guerin (BCG) vaccine at 5 but not 15 weeks of age protected from T1D (18) and injection of complete Freund’s adjuvant (CFA) protected from T1D only when given during early life (19). Similarly, antibiotic treatment of NOD mice during early life partially protected them from disease development in comparison to the treatment of adult NOD mice (20). While clearly an early-life immune stimulus seems to be important in the NOD mouse model, it remains unclear whether signals from the commensal microbiota during the early-life period would be sufficient to lower the disease incidence in NOD mice and to which extent this microbiota-mediated protection would be restricted to a certain phase of life.

Here, we used germ-free NOD mice that were exposed to SPF microbiota either at the time of birth or after weaning and followed their onset of T1D in combination with microbiota analyses and immunological assessments to evaluate the importance of early-life microbiota in the protection from auto-immune mediated T1D. This research underscores the potential of early-life microbial interventions in managing or even preventing autoimmune diseases.

## Results

### Colonization during birth, but not at weaning, confers protection against the development of type-1 diabetes in NOD mice

**Figure 1:**
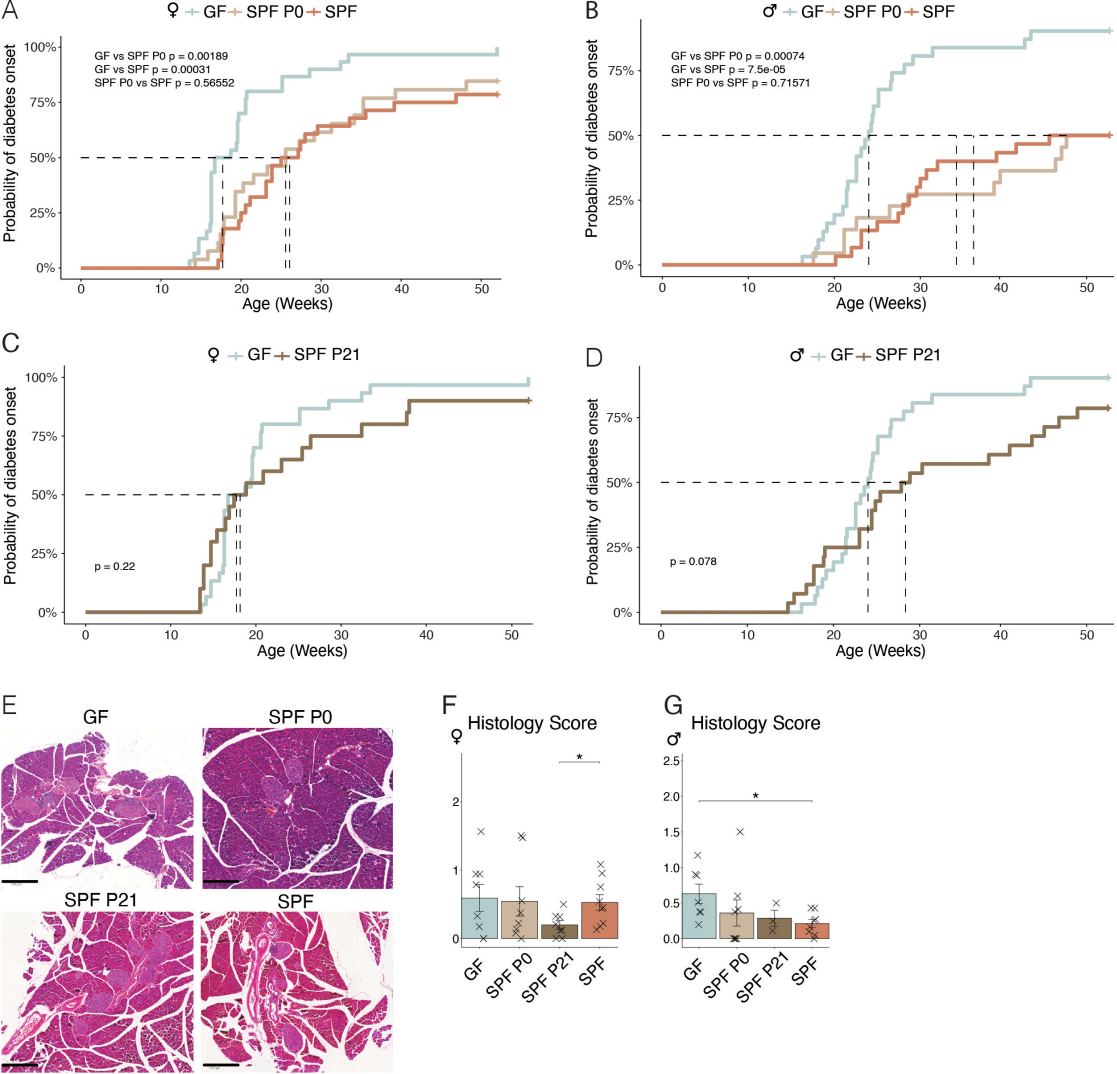
Colonization before weaning protects against the development of type-1 diabetes. Type-1 diabetes incidence was assessed by monitoring of non-fasted blood glucose levels in germ-free (GF), specific pathogen-free (SPF) NOD mice, and GF mice colonized at birth (SPF P0) or weaning (SPF P21), starting from 14 weeks and continuing until disease onset/up to 1 year of age. Cumulative disease incidence in **(A)** female and **(B)** male NOD mice, for GF compared to SPF, and SPF P0, respectively (n ≥ 28 per group). **(C)** Female and **(D)** male GF versus SPF P21 NOD mice disease incidence (n ≥ 29 per group). **(E)** Representative images of hematoxylin-eosin staining of the pancreata from GF, SPF P0, SPF P21, and SPF NOD mice at 8 weeks of age for insulitis scoring. Insulitis severity in **(F)** female and **(G)** male NOD mice (n = 8 per group). Unpaired parametric student’s t-test (*p ≤ 0.05) and mean ± standard deviation are shown.

Knowing that the spontaneous diabetes incidence in the NOD mouse model is highly variable between husbandries, we first characterized the cumulative disease incidence in our germ-free (GF) and specific pathogen-free (SPF) colonies by regularly measuring steady-state glucose levels in all mice until the occurance of a critical serum glucose level of 250 mg/dl, which was previously defined as diabetic state and the mouse subsequently euthanized (21). As expected, both female and male GF mice showed an earlier development of T1D, measured as the time passed until a cumulative disease incidence of 50%, compared to SPF mice (blue versus orange lines in **Figures 1A, B**). To address the importance of the microbiota during a particular time window in early life, we colonized newborn GF pups either at the time of birth (postnatal day P0) or at weaning (P21) with the SPF microbiota from our facility harboring the SPF NOD colony. Colonization was achieved by co-housing the dams and her newborn pups or the weaned offspring with a female SPF colonized mouse. While colonization at the time of birth (SPF P0) was able to delay the onset of T1D to a level that was equal to that observed in the SPF colony (beige line in **Figures 1A, B**), colonization at weaning (SPF P21) was unable to do so (**Figures 1C, D**), most evident in female SPF P21 NOD mice which showed the same disease susceptibility as their GF counterparts (**Figure 1C**). Moreover, SPF P0 tended to develop T1D later than SPF P21 mice (**Supplementary Figure 3**). A comparable delay in diabetes onset was observed in mice colonized at the day of birth with the stable defined moderately diverse mouse microbiota (sDMDMm P0), a less diverse gnotobiotic microbiota containg 12 bacterial species (**Supplementary Figure 1**) (22). Although male SPF P21 mice were slightly more protected from T1D development than GF male NOD mice, no significant difference was observed at the 50% cumulative disease incidence (**Figure 1D**). Regardless of the colonization status, female mice developed the disease earlier than male mice (**Figures 1A–D**; **Supplementary Figure 2**), which was in accordance with previous reports (12). Accordingly, female mice exhibited a higher insulitis score index at 8 weeks of age compared to male mice (**Figures 1E–G**). However, the insulitis score at this time point was inconsistent with the observed T1D incidence (**Figures 1A–D**). Although female SPF P21 mice exhibited a similar cumulative disease incidence as GF mice (**Figure 1C**), they harbored a significantly lower insulitis score than fully colonized SPF females, which were partly protected from the disease (**Figures 1A, F**). Similarly, in males the insulitis scoring (**Figure 1G**) did not match the cumulative disease incidence (**Figures 1B, D**), suggesting that the timing of onset of inflammatory lesions within pancreatic islets may not determine the ultimate development of T1D. Nevertheless, the protective effect of the commensal microbiota in the NOD model seems to be restricted to a limited time window between birth and weaning.

### Colonization at weaning restores intestinal barrier integrity but does not protect against type-1 diabetes

**Figure 2:**
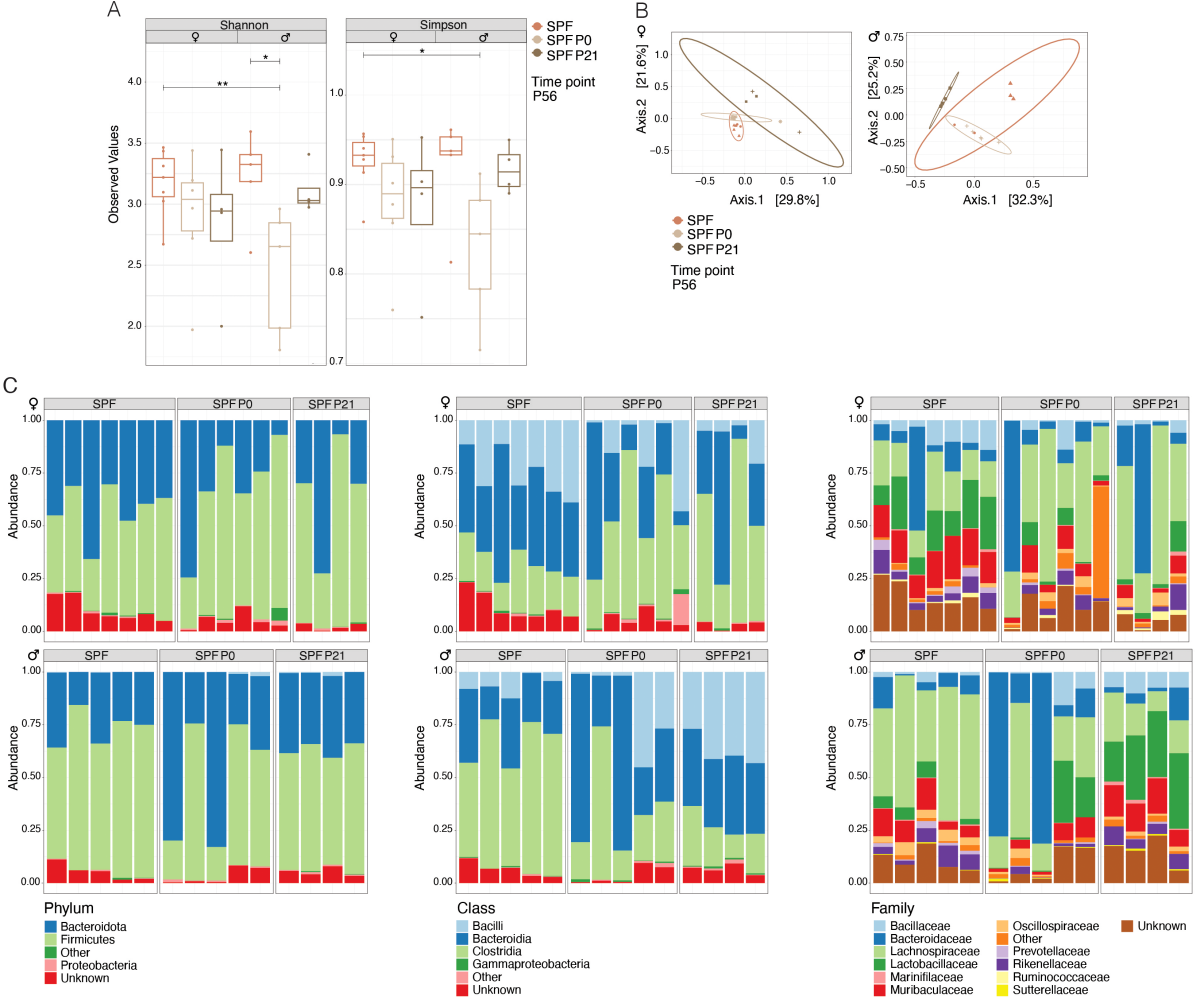
Colonization time point has no exert lasting effects on the composition of the microbiota. 16s rRNA sequencing was performed on fecal samples at postnatal day 56 (P56) from specific pathogen-free (SPF) and those colonized at birth (SPF P0) or at weaning (SPF P21) with the SPF microbiota. **(A)** *α*-diversity (Shannon and Simpson indices) for female and male NOD mice, respectively. Each symbol represents an individual mouse. **(B)** β-diversity based on Bray-Curtis dissimilarity for female and male NOD mice, respectively. Each symbol represents a different cage. **(C)** Relative abundance of bacteria at the phylum, class, and family levels in female and male NOD mice, respectively. One-Way ANOVA (*p ≤ 0.05, **p ≤ 0.01, n ≥ 4 per group) and mean ± standard deviation are shown.

Quantitative and qualitative differences in the commensal microbiota composition are one likely explanation for discrepant disease susceptibility in NOD mouse colonies around the world (14). We conducted 16S rRNA gene sequencing on fecal samples from 8 week-old mice. Our analysis revealed that neither α-diversity as assessed with Shannon and Simpson indices (**Figure 2A**) nor β-diversity using the Bray-Curtis dissimilarities matrix (**Figure 2B**) showed significant differences between mice raised under SPF conditions (SPF and SPF P0) and those colonized only at weaning (SPF P21). Taxonomic analysis revealed no major deviation in the microbiota composition at the phylum, class, or family level (**Figure 2C**; **Table 1**). Thus, persisting differences in intestinal microbiota composition are unlikely to explain the observed phenotypic variations in T1D onset. However, the absence of microbiota until P21 and the potential dysbiosis from colonization at weaning could influence immune regulation and intestinal barrier integrity over the long term in the P21 colonized group.

**Table 1:** Taxonomy Statistics comparing SPF P0 and SPF P21 using ASV.

Bacteria	Sex	Up in	coefficient	standard deviation	N	p-value	q-value
*c_Clostridia.o_Lachnospirales.f_Lachnospiraceae.g_ASF356*	F	SPF P21	0.07813282	0.028942065	10	0.02708962	0.65015091
*d_Bacteria.p_Bacteroidota.c_Bacteroidia._._._*	F	SPF P0	-0.0805548	0.062012737	10	0.23012869	0.83828862
*c_Bacteroidia.o_Bacteroidales.f_Prevotellaceae._*	F	SPF P0	-0.0464613	0.030968353	10	0.17193157	0.83828862
*c_Clostridia._._._*	F	SPF P21	0.05371798	0.041716342	10	0.23385044	0.83828862
*c_Clostridia.o_Lachnospirales.f_Lachnospiraceae.g_Lachnospiraceae_UCG.001*	F	SPF P21	0.20527358	0.153978611	10	0.21920197	0.83828862
*c_Clostridia.o_Oscillospirales.f_Ruminococcaceae._*	F	SPF P21	0.06252645	0.049776074	10	0.24450085	0.83828862
*c_Gammaproteobacteria.o_Burkholderiales.f_Sutterellaceae.g_Parasutterella*	F	SPF P0	-0.0413066	0.026428209	10	0.15668564	0.83828862
*c_Bacteroidia.o_Bacteroidales.f_Muribaculaceae.g_Muribaculaceae*	F	SPF P0	-0.1007293	0.088296928	10	0.28695038	0.86085114
*c_Bacteroidia.o_Bacteroidales._._*	F	SPF P0	-0.0506274	0.072868526	10	0.50686832	0.89000887
*c_Bacteroidia.o_Bacteroidales.f_Rikenellaceae._*	F	SPF P0	-0.0383832	0.044219879	10	0.41067115	0.89000887
*c_Bacteroidia.o_Bacteroidales.f_Rikenellaceae.g_Rikenellaceae_RC9_gut_group*	F	SPF P0	-0.0424424	0.045644506	10	0.37965328	0.89000887
*c_Clostridia.o_Lachnospirales.f_Lachnospiraceae._*	F	SPF P0	-0.1017865	0.150968172	10	0.51917184	0.89000887
*c_Clostridia.o_Lachnospirales.f_Lachnospiraceae.g_Lachnospiraceae_NK4A136_group*	F	SPF P21	0.03796666	0.037682493	10	0.3431736	0.89000887
*c_Clostridia.o_Oscillospirales.f_Oscillospiraceae._*	F	SPF P0	-0.0463007	0.059699222	10	0.46031438	0.89000887
*c_Clostridia.o_Lachnospirales.f_Lachnospiraceae.g_Lachnoclostridium*	F	SPF P21	0.0378152	0.068197202	10	0.59439612	0.89159419
*c_Alphaproteobacteria.o_Rickettsiales.f_Mitochondria.g_Mitochondria*	F	SPF P0	-0.0249126	0.040912825	10	0.55946765	0.89159419
*c_Bacteroidia.o_Bacteroidales.f_Bacteroidaceae.g_Bacteroides*	F	SPF P0	-0.0003613	0.223207185	10	0.99874825	0.99874825
*c_Bacteroidia.o_Bacteroidales.f_Marinifilaceae.g_Odoribacter*	F	SPF P21	0.0143678	0.037205592	10	0.70943597	0.99874825
*c_Bacteroidia.o_Bacteroidales.f_Muribaculaceae.g_Muribaculum*	F	SPF P0	-0.0074583	0.032910063	10	0.82639828	0.99874825
*c_Bacteroidia.o_Bacteroidales.f_Rikenellaceae.g_Alistipes*	F	SPF P0	-0.0107345	0.073531304	10	0.88754385	0.99874825
*c_Bacilli.o_Bacillales.f_Bacillaceae.g_Bacillus*	F	SPF P0	-0.0092644	0.095418128	10	0.92504118	0.99874825
*c_Bacilli.o_Lactobacillales.f_Lactobacillaceae.g_Lactobacillus*	F	SPF P21	0.00136118	0.099304534	10	0.98939932	0.99874825
*c_Clostridia.o_Lachnospirales.f_Lachnospiraceae.g_A2*	F	SPF P21	0.00734277	0.072164233	10	0.92145852	0.99874825
*c_Clostridia.o_Oscillospirales.f_Butyricicoccaceae._*	F	SPF P0	-0.0151719	0.058468417	10	0.80181128	0.99874825
*c_Bacteroidia.o_Bacteroidales.f_Rikenellaceae.g_Rikenellaceae_RC9_gut_group*	M	SPF P21	0.13962254	0.013463694	9	1.68E-05	0.00040382
*c_Bacteroidia.o_Bacteroidales.f_Marinifilaceae.g_Odoribacter*	M	SPF P21	0.09682504	0.020003311	9	0.00187678	0.02252142
*c_Clostridia.o_Lachnospirales.f_Lachnospiraceae.g_Lachnospiraceae_NK4A136_group*	M	SPF P21	0.17895048	0.051935438	9	0.01075476	0.08447468
*c_Clostridia.o_Oscillospirales.f_Oscillospiraceae._*	M	SPF P21	0.08784024	0.027037103	9	0.01407911	0.08447468
*c_Bacilli.o_Lactobacillales.f_Lactobacillaceae.g_Lactobacillus*	M	SPF P21	0.38430922	0.144646862	9	0.03261447	0.13045787
*c_Clostridia.o_Lachnospirales.f_Lachnospiraceae.g_Lachnospiraceae_UCG.001*	M	SPF P0	-0.3397671	0.122329345	9	0.02739751	0.13045787
*c_Bacilli.o_Erysipelotrichales.f_Erysipelotrichaceae.g_Dubosiella*	M	SPF P21	0.05774211	0.023346102	9	0.04262588	0.14614587
*c_Bacteroidia.o_Bacteroidales.f_Rikenellaceae._*	M	SPF P0	-0.0393884	0.018234133	9	0.06759357	0.20278071
*c_Bacteroidia.o_Bacteroidales.f_Bacteroidaceae.g_Bacteroides*	M	SPF P0	-0.4004022	0.223177303	9	0.11588075	0.29131754
*c_Gammaproteobacteria.o_Burkholderiales.f_Sutterellaceae.g_Parasutterella*	M	SPF P21	0.04105586	0.023295879	9	0.12138231	0.29131754
*c_Clostridia.o_Lachnospirales.f_Lachnospiraceae._*	M	SPF P0	-0.1136102	0.067930888	9	0.13835676	0.3018693
*c_Bacteroidia.o_Bacteroidales._._*	M	SPF P21	0.09681411	0.062655748	9	0.16622004	0.30686776
*c_Bacteroidia.o_Bacteroidales.f_Muribaculaceae.g_Muribaculaceae*	M	SPF P21	0.15128946	0.097642229	9	0.16521086	0.30686776
*c_Bacteroidia.o_Bacteroidales.f_Rikenellaceae.g_Alistipes*	M	SPF P21	0.06610467	0.054860492	9	0.26737011	0.42779217
*c_Clostridia._._._*	M	SPF P21	0.04599855	0.036940618	9	0.25312249	0.42779217
*c_Clostridia.o_Lachnospirales.f_Lachnospiraceae.g_A2*	M	SPF P21	0.05556244	0.048604412	9	0.29055899	0.43583848
*c_Clostridia.o_Lachnospirales.f_Lachnospiraceae.g_ASF356*	M	SPF P21	0.03546573	0.036654069	9	0.36548741	0.51598223
*c_Bacteroidia.o_Bacteroidales.f_Muribaculaceae.g_Muribaculum*	M	SPF P21	0.02714728	0.037909949	9	0.49711771	0.59654125
*c_Bacilli.o_Bacillales.f_Bacillaceae.g_Bacillus*	M	SPF P21	0.07606773	0.100381901	9	0.47331427	0.59654125
*c_Clostridia.o_Lachnospirales.f_Lachnospiraceae.g_Lachnoclostridium*	M	SPF P0	-0.057322	0.072030744	9	0.45228614	0.59654125
*c_Clostridia.o_Oscillospirales.f_Ruminococcaceae._*	M	SPF P0	-0.022911	0.042858913	9	0.60950062	0.69657213
*c_Actinobacteria.o_Bifidobacteriales.f_Bifidobacteriaceae.g_Bifidobacterium*	M	SPF P21	0.02027486	0.044605993	9	0.66320559	0.72349701
*c_Bacteroidia._._._*	M	SPF P0	-0.0142216	0.040140054	9	0.73354802	0.76544141
*c_Bacteroidia.o_Bacteroidales.f_Prevotellaceae*	M	SPF P0	-0.008181	0.029126079	9	0.78692259	0.78692259

**Figure 3:**
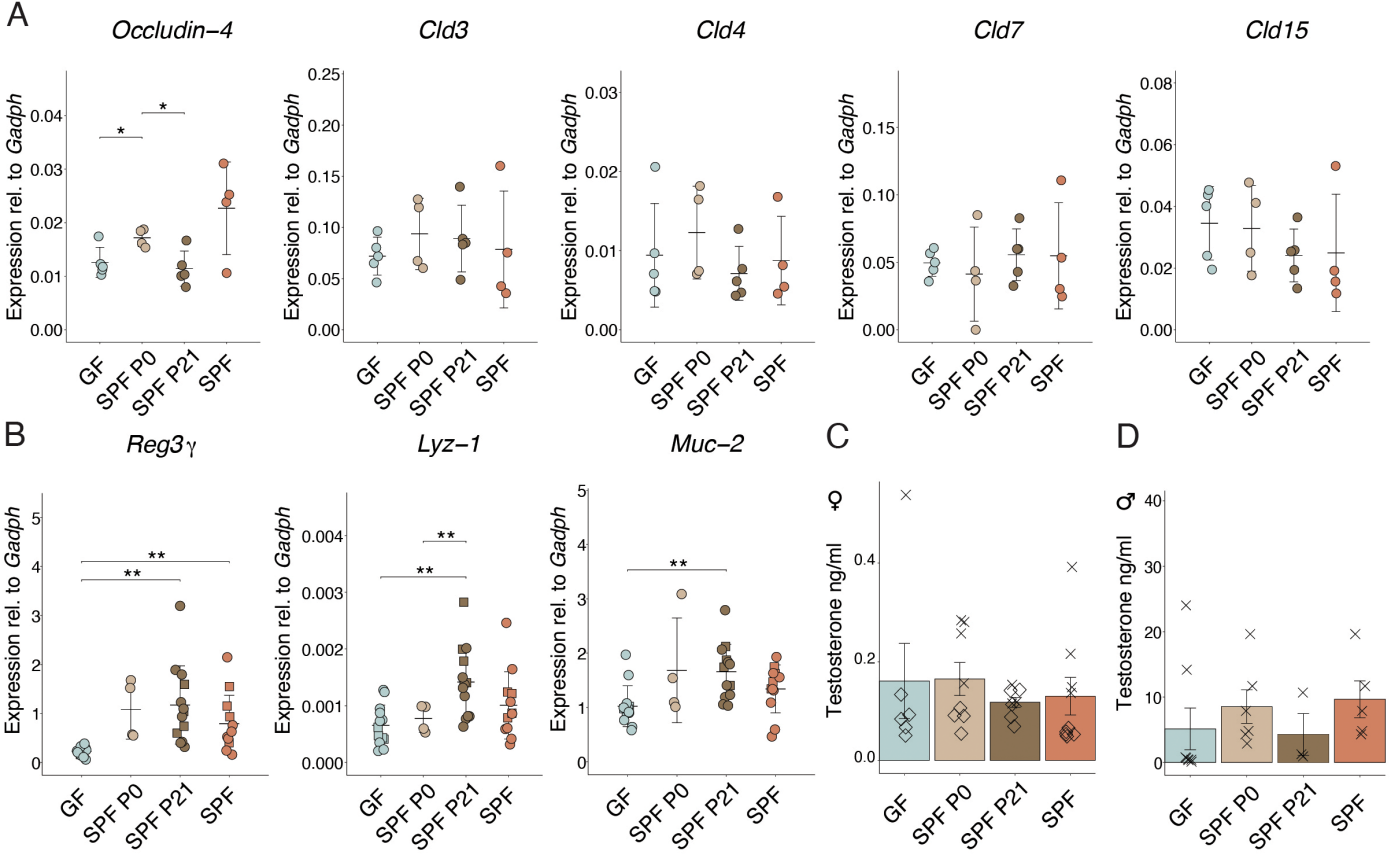
Colonization alters gene expression in the small intestine. **(A)** The expression of *Occludin-4,Cld3, Cld4, Cld7, Cld15*, and **(B)** *Reg3*γ*, Lyz-1, and Muc-2* genes in the ileum were determined at postnatal day 56 in GF, SPF, SPF P0, and SPF P21 mice by RT-qPCR, and normalized to *Gapdh* (n > 4 per group). Males are depicted as squares, whereas females are represented as dots. Testosterone levels were measured in the serum of **(C)** female and **(D)** male NOD mice, respectively (n ≥ 3 per group). Each symbol represents a different animal, while different symbols (cross/diamond) represent animals from independent experiments. Plots show pooled data from two **(C, D)** independent experiments, respectively. Unpaired parametric student’s t-test (*p ≤ 0.05, **p ≤ 0.01, ***p ≤ 0.001) and mean ± standard deviation are shown.

Enhanced intestinal permeability was shown to occur before insulitis and T1D in animals and humans (23–26). No differences in the expression of genes encoding for claudin molecules or in Occludin-4, all of which encode for tight junction components, could be observed between the groups (**Figure 3A**), suggesting that the time point of colonization does not permanently shape the intestinal barrier integrity. GF mice expressed lower RNA levels of *Reg3g*, encoding for antimicrobial peptides compared to SPF mice (**Figure 3B**). *Reg3g, Lyz-1, and Muc-2* protein products additionally contribute to a functional intestinal barrier. Interestingly, adult SPF P21 NOD mice did not exhibit any sign of an impaired intestinal barrier compared to the SPF counterparts (**Figures 3A, B**), indicating that microbiota colonization at weaning time frame is sufficient for maintaining intestinal barrier integrity and but not for protection against the onset and progression of T1D.

### Early life colonization modulates testosterone levels and type-1 diabetes susceptibility in male NOD mice

A clear difference in T1D susceptibility between GF and SPF mice was shown by Danska and colleagues (13). Remarkably, the protective effect of the SPF microbiota was attributed to the level of male steroid hormones in the colonizing microbiota. Colonization of adult GF NOD mice with a male microbiota lowered diabetes susceptibility, while colonization with a female microbiota did not confer any protection. In our model, we invariably colonized GF mice using a female microbiota. Consequently, the previously published data are in line with our observations, where colonization with a female microbiota, as used in our case, in adult life was not protective. Similarly, we measured the sex hormone levels in the serum of adult (8-week-old) NOD mice in all our experimental groups. As expected, female mice harbored substantially low testosterone levels (between 0-0.5 ng/ml). Results showed slightly higher testosterone levels in GF and SPF P0 compared to SPF P21 and SPF females (**Figure 3B**). Interestingly, males colonized at the time of birth or born from an SPF-colonized mother exhibited slightly higher levels than GF mice and those colonized with an SPF microbiota at weaning (**Figure 3C**). This result indicates that earlier colonization with a microbiota might increase steroid hormone levels in male mice and underscores the previously published contribution of the microbiota to this process. While the presumable microbiota-induced sex hormone induction seems to partially explain the phenotype observed in the P0 versus P21 colonized male NOD mice, it cannot account for the differences described in the female colonies.

### Colonization induces changes in T cell subset frequencies within the small intestine, influencing type-1 diabetes development in NOD mice

T1D is an autoimmune disease in which auto-reactive T cells mediate the destruction of *β* cells in the pancreatic islets. A dysbalance between effector (Teffs) and regulatory T cells (Tregs) was suggested to be one of the causes for disease development (27). Thymic-derived naturally occurring CD25^+^CD4^+^ regulatory T (nTregs) cells have been shown to have protective effects in both the NOD animal model as well as in humans. Evidence from numerous studies confirmed that absence of CD25^+^ T cells in mice facilitated the development of T1D, while adoptive transfer of these cells or IL-2 supplementation resulted in the resolution of the diabetes symptoms (28–33).

**Figure 4:**
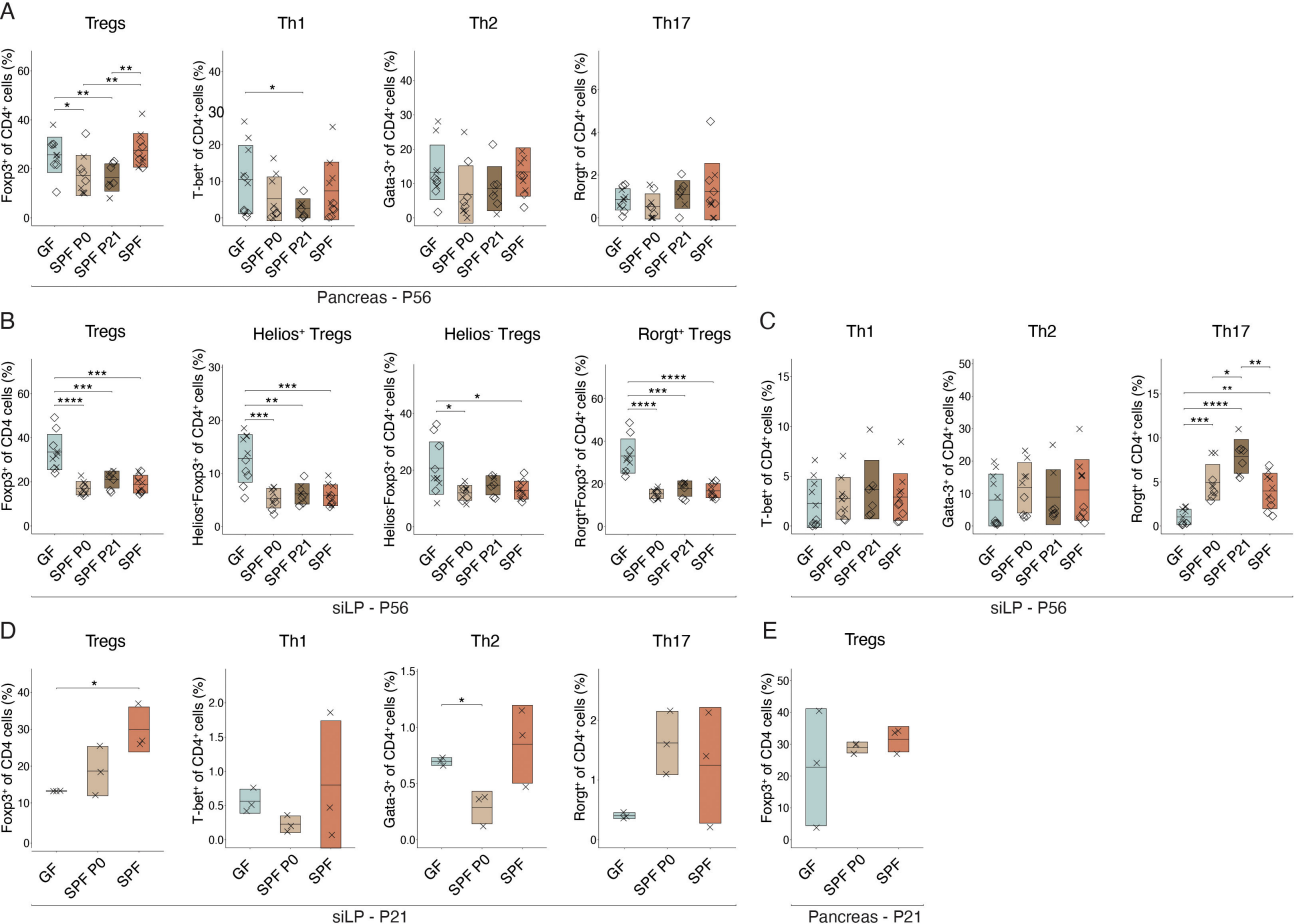
Intestinal colonization affects T helper cell frequencies in the small intestine. Single-cell suspensions were obtained from pancreas or small intestinal lamina propria (siLP) at postnatal day 56 (P56) (n ≥ 7 per group) or 21 (P21) (n = 3 per group) and analyzed by flow cytometry. Frequency of regulatory T cells (Tregs), Th1, Th2, and Th17 cells **(A)** in the pancreas at P56 and **(D)** in the siLP at P21. Frequency of **(B)** Tregs, subdivided into Helios^+^, Helios^+^, Rorgt^+^, and Rorgt^-^ Tregs in the siLP at P56, **(C)** Th1, Th2, Th17 cells in the siLP at P56, and **(E)** Tregs in the pancreas at P21, respectively. Unpaired parametric student’s t-test (*p ≤ 0.05, **p ≤ 0.01, ***p ≤ 0.001, ****p ≤ 0.0001, n ≥ 3 per group) and mean ± standard deviation are shown. Symbols indicate individual experiments. Each symbol represents a different animal, while different symbols (cross/diamond) represent animals from independent experiments. Plots show pooled data from two **(A-C)** or one **(D, E)** independent experiments, respectively.

The pancreas-infiltrating lymphocytes in the NOD model may originate and migrate from gut-associated lymphatic tissues (GALT) in the intestine to the pancreas, as demonstrated by the expression of gut-homing markers, such as α4β7 integrin (34). Therefore, we analyzed T cell populations in the pancreas of pre-diabetic 8 week-old mice in all experimental groups by flow cytometry. No difference coherent with experimental groups in Teff or Treg cell frequencies were observed between GF and any colonized SPF females in the pancreatic leukocytes (**Figure 4A**). Remarkably in the small intestinal lamina propria (siLP), Treg proportions were significantly lower in all colonized animals, regardless of the colonization time point, compared to GF controls. Further analysis revealed that the decrease was not restricted to natural (Helios^+^) or induced (Rorgt^+^/Helios^-^) Tregs, but present in both subsets (**Figure 4B**). Interestingly, no differences in Th1 and Th2 cells were observed. However, colonized animals harbored higher frequencies of Th17 cells in the siLP compared to GF mice (**Figure 4C**).

Homing of α4β7-expressing lymphocytes into the pancreas was shown to occur from week 3 post birth (34). In addition, protective nTregs and autoreactive Teff seem to be present as early as 3 weeks of age (27, 30). Hence, we wondered whether differences in organ distribution of T cell populations at this early age may be visible and possibly explain the distinct T1D susceptibility observed in NOD mice colonized at birth or weaning. Flow cytometric analysis at P21 confirmed a difference in Treg frequencies in the siLP between GF and SPF colonized mice with relatively higher Treg propotions in SPF and P0 colonized compared to GF mice (**Figure 4D**). At this time point, SPF P21 were excluded from the analysis as they were still GF. Other T cell populations and pancreatic Treg proportions were not significantly affected by microbial colonization at this early time point (**Figure 4E**).

Lastly, to determine whether a certain distribution among CD4^+^ T cell subsets prior to weaning in SPF mice contribute to the microbiota-dependent protective effect against diabetes, we depleted all CD4^+^ T cells from SPF pups between the age of 10 to 20 days using injections of depleting anti-CD4 antibodies or isotype control antibodies (**Supplementary Figure 5C**). Despite this intervention, both female and male mice that underwent CD4^+^ T cell depletion exhibited a cumulative disease incidence comparable to their control counterparts. This suggests that the complete absence of T helper cells in early life does not critically influence T1D pathogenesis. Instead, it appears that altered proportions of T helper cell subsets during this period may promote the development of the disease (**Supplementary Figures 5A, B**).

## Discussion

Our study provides compelling evidence that the timing of microbial colonization significantly influences the development of T1D in NOD mice, supporting the concept that specific early-life windows are critical for establishing a regulatory mechanisms that can modulate disease risk. These findings substantiate theories like the hygiene hypothesis and the disappearing microbiota hypothesis, which correlate diminished microbial diversity with an increase in autoimmune diseases (8, 9, 35, 36).

Additionally, our data align with previous research suggesting that reduced microbial diversity in early life escalates the risk of T1D, underscoring the enduring impact of early-life microbial environments on host immune modulation and disease susceptibility (37, 38). Microbiota development mirrors the development of the immune system in distinct waves during early life, but while external factors persistently impact the immune system, visible changes in the microbiota diminish with age (39–42). Our findings show no differences in the composition of the adult microbiota between the groups (**Figures 2A–C**), indicating that the microbiota composition at or directly before disease onset likely does not explain the observed T1D incidence. Instead, intestinal colonization at distinct early lifetime points, coinciding with phases of immune system development, may account for the differences in T1D predisposition in NOD mice. Interestingly, colonization at P0 with the less diverse sDMDMm microbiota was sufficient to confer a delay in the onset of T1D, indicating that species richness alterations before weaning were not the driving factor in our phenotype (**Supplementary Figure 1**). Accordingly, early-life colonization of NOD mice with the microbiota of disease-protected C57BL/6 mice did not confer protection against T1D (43), supporting the notion that the composition of the microbiota does not necessarily determine the disease susceptibility. Nevertheless, another study recently showed that the transfer of a specific microbiota protects NOD mice against the developmend of T1D (44), indicating that a combination of distinct factors, including a specific microbial consortia and higher microbial diversity (37, 38) may shape the predisposition to T1D. Furthermore, our data implies that colonization of the mother before giving birth seems to be neglectable or rescued by postnatal colonization of the offspring itself in our model. We did however not test the influence of exclusive colonization of the dam during pregnancy with the reversible auxotrophic *E. coli* model (45) on disease susceptibility in the resulting offspring.

Our results revealed elevated expression of genes related to the intestinal barrier function in adult colonized compared to GF NOD mice (**Figure 3B**), irrespective of colonization time point. Notably, increased intestinal permeability, linked to T1D risk, may result from a leaky gut allowing dietary antigen passage, triggering immune reactions and fascilitating T1D development (46). However, colonization at weaning induced expression of genes encoding for proteins with gut barrier function similar to SPF NOD mice in adulthood (**Figure 3B**). Nevertheless, SPF P21 carry a history of transiently increased permeability before weaning when not yet colonized, as observed in GF mice throughout life, potentially influencing T1D susceptibility later in life. The expression of genes encoding tight junction proteins was comparable between the groups at P56 (**Figure 3A**), indicating that colonization at weaning does not lead to a permanent perturbation of the intestinal barrier.

Disease susceptibility in NOD mice significantly differs between genders (47). Castration increased T1D incidence in NOD males, highlighting the protective role of male hormones (48). Androgens influence gut microbiota composition, protecting against T1D (49), while the microbiota modulates androgen metabolism (50). Fecal microbiota transplantation (FMT) from male, but not female, donors protects adult NOD mice against T1D, attributed to testosterone levels in the colonizing microbiota (13). In our experiments, colonization with a female microbiota at birth or weaning resulted in different disease onsets (**Figures 1A–D**), possibly due to microbiota evolving into an “adult male” microbiota before weaning in males, while this capacity may have disappeared by weaning, leading to disease onset similar to GF mice. Correspondingly, slightly higher testosterone levels were found in SPF and SPF P0 compared to GF and SPF P21 NOD males (**Figure 3D**), suggesting earlier colonization increases androgen levels in male NOD mice. However the opposite phenotype was observed in females, with slightly higher testosterone levels in GF compared to SPF P21 and SPF females (**Figure 3C**). Accordingly, lower androgen levels in colonized compared to GF NOD females have been published before (13). Thus, while microbiota-induced sex hormone induction partly explains the observed phenotype in SPF P0 versus SPF P21 males, it may not fully account for the differences in female colonies.

The microbiota plays a crucial role in the differentiation of T helper cells in the intestine, associated with immune-mediated diseases like T1D. Initial assumptions regarding Th1 cells driving T1D development in NOD mice (51) were challenged by evidence showing that Th2 cells can induce pancreatitis (52). The general assumption now refers to a Th1/Th2 cell imbalance contributing to T1D development (27, 47, 53). The microbiota drives Th1 development, confirming its role in balancing Th1/Th2 responses (54). Beyond Teffs, Tregs are critically involved in T1D pathogenesis, with their development being equally dependent on intestinal colonization (55–57). This implies that the microbiota may regulate T helper cell versus Treg balances, thereby modulating T1D susceptibility.

Investigating the extent to which early life microbiota alters the T cell compartment in female colonies, we found distinct CD4^+^ T cell subsets in the siLP of adult NOD mice depending on microbial colonization (**Figures 4B, C**). Fittingly, discrepancies in Treg and Th17 cell frequencies between GF and SPF mice have been detected in mesenteric and pancreatic lymph nodes and linked to an enhanced insulitis development (58). Notably, Treg frequency was significantly higher in adult siLP of GF compared to colonized mice, while the Th17 cell proportion was elevated in colonized compared to GF mice (**Figures 4B, C**), supporting the notion of the ability of the microbiota in affecting T cell subset balances in the intestine. However, the frequency of Tregs and Teffs were comparable between all groups of colonized NOD females, indicating a time-independent effect of the microbiota in shifting T cell proportions present in adult NOD mice. However, 3 week-old GF females exhibited distinct Treg frequencies in the siLP compared to SPF females (**Figure 4D**), indicating an early influence of the microbiota on the Treg and Teff equilibrium, potentially impacting T1D predisposition.

At 3 weeks of age, diabetogenic T cells, which are primed in gut-associated lymphatic tissues, migrate into the pancreas (34). Around this time, GF mice, which at this time point include also the later colonized P21 group, exhibited significantly lower levels of Tregs in the siLP compared to colonized animals (**Figure 4D**), in line with the knowledge of microbiota-induced Tregs (56). Comparable pancreatic frequencies of Tregs and Teffs between GF and SPF females (**Figures 4A, E**), suggest that a distinct functionality may determine T1D susceptibility in NOD females, possibly induced by an early-life shift in Treg versus Teff numbers. Accordingly, Treg functionality was reduced in GF mice (57). Interestingly, Tregs generated in early life are functionally distinct from those generated in adulthood (59). Thus, early life microbiota-induced Tregs may possess superior suppressive functionality, influencing disease predisposition.

Complete deletion of CD4^+^ T cells during early life did not impact T1D incidence in SPF NOD mice (**Supplementary Figures 1B, C**), emphasizing the complex role of T cells, particularly of the Treg and Teff balance in disease onset. Remarkably, thymectomy around weaning, eradicating both diabetogenic and protective T cells, accelerated T1D development (60). Similarly, CD4 depletion eliminates all T helper cells alongside Tregs, explaining why this procedure did not protect against T1D development. Hence, a disequilibrium of Teffs and Tregs during early life and a distinct functionality of Tregs due to the absence of microbial colonization during early life most probably contributes to T1D susceptibility in GF and SPF P0 colonized NOD mice.

In conclusion, our data support the notion that differential priming in the intestine during early life windows may contribute to variations in T1D development between mice colonized earlier or later in life.

### Limitations of the study

Our results revealed a higher insulitis score index at 8 weeks of age in females compared to males, which was consistent with their earlier T1D development (**Figures 1A–G**; **Supplementary Figure 2**). However, the insulitis score of the different colonies at this time point did not align with the observed T1D development (**Figures 1A–D**). Female SPF P21 and GF mice harbored similar cumulative disease incidences (**Figure 1C**), yet their insulitis scores were distinct (**Figures 1A, F**). Similarly, in males, the insulitis score index (**Figure 1G**) was inconsistent with the cumulative disease incidence (**Figures 1B, D**). This discrepancy suggests that the time point for insulitis scoring at 8 weeks of age may not be suitable to predict the time point of disease development. Therefore, a later time point should have been analyzed. This discrepancy in time frames is a limitation of the study, potentially explaining why the diabetes incidence and insulitis rates do not correlate.

Our results indicate only a trend toward earlier T1D development in the direct pairwise comparison of SPF P21 and SPF P0 mice. However, our data clearly demonstrate that colonization at birth (SPF P0) provided significant protection against T1D compared to GF mice, whereas colonization at weaning (SPF P21) did not confer the same level of protection. This is the key point we aim to highlight in this study and the absence of significances in the other comparison may be due to insufficient animal numbers in the individual groups. A relatively large number of animals was already analyzed and due to the fact that we analyze both females and males, we did not consider a further increase in animal numbers ethically justified to answer the research question.

Our data indicate a correlation between early colonization and higher testosterone levels in male NOD mice, which may partly explain the difference in disease onset observed between SPF P0 and SPF P21 males. However, we recognized that these trends do not meet the threshold for statistical significance. We also observed a trend of slightly higher testosterone levels in GF females compared to SPF P21 and SPF females, which aligns with the previously published (13). This suggests that microbiota-induced changes in sex hormone levels might contribute to the observed differences in disease onset, although this effect appears more complex and may not fully account for the differences our the colonies.

Last, despite claiming to have seen no clear evidence for an altered intestinal microbiota composition between the SPF P0 and SPF P21 groups, we did observe that two ASVs were significantly enriched in the microbiota of SPF P21 compared to the SPF P0 groups in male but not in female mice. We did not test if the addition of taxa of these two genera could accelerate diabetes onset in SPF P0 mice, nor did we not analyze for any difference in the presence of strain or substrains in the different experimental groups by metagenomic sequencing.

## Materials and methods

### Mice

Germ-free and gnotobiotic NOD/ShiLtJ mice were bred and maintained in flexible-film isolators in the Clean Mouse Facility, University of Bern as previously described (61). Germ-free status was regularly monitored by aerobic and anaerobic culture and SYTOX Green (Invitrogen) to stain nucleic acids, and thus detect potential bacterial and fungal contaminants in feces and bedding. All mice were housed in a 12 h-12 h light-dark cycle and received an autoclaved standard chow diet as well as autoclaved water.

SPF NOD/ShiLtJ mice were bred and maintained in individually-ventilated IVC cages in the Central Animal Facility, University of Bern. SPF mice were housed in a 12 h-12 h light-dark cycle and received an irradiated standard chow diet as well as autoclaved water. All animal experiments were performed in accordance with the Swiss Federal regulations on the approved experimental license BE36/2021.

### CD4 depletion in NOD mice

CD4^+^ T cells were depleted in NOD mice before weaning by administering an anti-CD4 antibody (clone GK5.1, Bioexcel) or an isotype control antibody (clone LTF-2, Bioexcel) on postnatal days 10, 13, 16, 18, and 20. 100 or 200 µg of antibodies diluted in PBS were injected intraperitoneally for the first three and last two doses, respectively.

### Manipulation of the intestinal microbiota

Germ-free mice were colonized at birth or weaning (P0, P21) with a model microbiota (sDMDMm (22) or SPF) by co-housing with a female colonizer for 14 days. The sDMDMm microbiota can be visualized in **Table 2**.

**Table 2:** Composition of the sDMDMm microbiota.

Species
*Lachnoclostridium sp.* YL32
*Ruminiclostridium sp.* KB18
*Enterococcus faecalis* KB1
*Bacteroides sp.* I48
*Burkholderiales bacterium* YL45
*Erysipelotrichaceae bacterium* I46
*Blautia sp.* YL58
*Parabacteroides sp.* YL27
*Flavonifractor plautii* YL31
*Bifidobacterium animalis subsp. animalis* YL2
*Lactobacillus reuteri* I49
*Akkermansia muciniphila* YL44

### Type-1 diabetes assessment in NOD mice

Blood glucose levels were measured every second week in non-fasted animals starting at 14 weeks until disease onset or 52 weeks of age using a glucometer. Mice were considered diabetic when their blood glucose levels reached 250 mg/dl. Statistical analysis was performed by Kaplan-Meier curves to estimate the probability of disease onset using RStudio (Version 2023.06.0 + 421).

### RNA isolation

1 cm of terminal ileum was collected and TRIzol (Invitrogen). Tissue was homogenized using a tissue lyser (Retsch^®^ MM400) for 3 min at 25 Hz. 200 µl of chloroform was added and samples were centrifuged at 12,000 g, 15 min, 4°C. The upper colorless aqueous phase was precipitated with 500 µl of ice-cold isopropanol and recentrifuged at 12,000 g, 10 min, 4°C. Samples were washed with 1 ml of ice-cold 75% ethanol and centrifuged at 7,500 g, 5 min, 4°C. The pelleted RNA was air-dried for 10 min and resuspended in RNase-free water (Gibco). The genomic DNA was digested DNase I (RNase-Free DNase Set, QIAGEN) according to manufacturer’s protocol. RNA samples were purified using the RNeasy MinElute Cleanup Kit (Qiagen) according to the manufacturer’s instructions and used for subsequent quantitative real-time polymerase chain reaction (RT-qPCR).

### Reverse transcription and quantitative real-time polymerase chain reaction

RNA concentration was measured using a NanoDrop2000 Spectrophotometer (Thermo Fisher Scientific™), cDNA synthesis was performed, by mixing 2 µg of RNA), 250 ng/µl random primers (Promega), 10 mM dNTPs in a total volume of 13 µl of water. After an incubation step of 5 min at 65°C, 4 µl 5X First-Strand Buffer (Invitrogen) and 1 µl Superscript III reverse transcriptase enzyme (Invitrogen), 1 µl o 0.1 M DTT (Invitrogen) and 1 µl of RNaseOUT (Invitrogen) were added and the reaction reverse transcription reaction was performed by incubating the samples at 55°C for 50 min. An additional step at 70°C for 15 min was performed followed by cooling at 4°C.

The RT-qPCR reactions were performed in triplicates using 25-50 ng of RNA in a total volume of 4 µl. 5 µl of SYBR Green (Qiagen), and 0.5 µl gene specific forward as well as reverse primer (10 µM) were added to the mixture. The mixture was initially heated to 95°C for 3 min, then 40 consecutive cycles of 95°C for 30 sec followed by 58°C for 30 sec were performed. The expression of the gene of interest (GOI) was calculated relative to the expression of a housekeeping gene (*Gapdh*) using the formula: 2^-Cycle threshold(GOI)-Cycle threshold(^ *^Gapdh^* ^)^. Primer sequences can be visualized in **Table 3**.

**Table 3:** Primer sequences for RT-qPCR.

Gene	Forward primer	Reverse primer
*Gapdh*	5’ CATCAAGAAGGTGGTGAAGC 3’	5’ CCTGTTGCTGTAGCCGTATT 3’
*Lyz-1*	5’ CTTGTCACTCCTCACCCCTG 3’	5’ AGCCGTTCCCCTTCCAATG 3’
*Muc-2*	5’ GCTGACGAGTGGTTGGTGAATG 3’	5’ GATGAGGTGGCAGACAGGAGAC 3’
*Occludin-4*	5’ TTGAAAGTCCACCTCCTTACAGA 3’	5’ CCGGATAAAAAGAGTACGCTGG 3’
*Reg3-γ*	5’ TTCCTGTCCTCCATGATCAAAA 3’	5’ CATCCACCTCTGTTGGGTTCA 3’

### DNA isolation

DNA isolation from 1-2 fecal pellets was performed using QIAamp PowerFecal Pro DNA Kit (Qiagen) according to the manufacturer’s protocol. Extracted DNA was stored at -20°C until further processing.

### 16s rRNA sequencing

3-6 µl of DNA, 12.5 µl Kapa Hifi enzyme (Invitrogen), and 1 µl forward as well as reverse primers to amplify the variable region V4 of 16S rRNA genes according to the manufacturer’s protocol on a C1000™ touch Thermal Cycler (Biorad). For PCR, barcoded forward primers (5’-CCATCTCATCCCTGCGTGTCTCCGACTCAG BARCODE GTGCCAGCMGCCGCGGTAA-3’) were used, where the core was modified by the addition of a PGM sequencing adaptor, a GT-spacer and a unique barcode that allowed to pool up to 96 different barcodes in combination with the reverse primer (5’-CCTCTCTATGGGCAGTCGGTGAT GGACTACHVGGGTWTCTAAT-3’) (62, 63).

The amplicon PCR reaction was performed with an initial heating step at 94°C for 5 min, followed by 35 cycles of denaturation at 94°C for 1 minute, annealing at 46°C for 20 sec, and extension at 72°C for 30 sec. The reaction concluded with a final extension at 72°C for 7 minutes and was then cooled to 4°C. Amplified PCR products were separated in a 1.5% agarose gel electrophoresis and purified using the Qiaquick Gel Extraction Kit (Qiagen). The concentration was measured using a Qubit 3.0 Fluorometer (Thermo Fischer). Uniquely barcoded samples were diluted (26 pM) and up to 96 samples were pooled for the library. Libraries were prepared with the OT2 HiQ View 400 kit for up to 400 bp reads, and emulsion PCR was performed on the Ion OneTouch 2 (OT2) instrument (Thermo Fischer). To generate Ion PGM™ Template OT2 400 Ion Sphere™ Particles (ISPs) that contain clonally amplified DNA, we utilized the Ion OneTouch™ Instrument along with the Ion PGM™ Template OT2 400 Kit provided by the manufacturer (Thermo Fisher). Subsequent sequencing was conducted using the Ion PGM™ Sequencing 400 Kit and the Ion 316™ Chip V2, all within the Ion PGM™ System (Thermo Fisher) (64).

Raw sequences were initially processed using the QIIME2 pipeline on the UBELIX Linux cluster at the University of Bern, where a Q-score-based trimming and filtering strategy was employed. After the sequences underwent quality checks and chimera removal within the DADA2 framework (65), only samples yielding over 2000 high-quality reads were selected for advanced analysis using various R packages.

Sequences with a Q-score over 30 proceeded to downstream analyses, including amplicon sequence variant (ASV) generation with 100% identity. We generated table.qza and rep-seq.qza files which were then used for taxonomic classification. Following sequence processing, taxonomic classification was conducted using a pre-trained classifier from the SILVA 132 database, available at https://docs.qiime2.org/2018.8/data-resources/, applied via the feature-classifier *classify-sklearn* command (66). To analyze the taxonomic composition and prevalence within each sample, as well as to determine the proportion of reads per taxonomic level, the taxonomy.qza file was converted into a bar graph using the taxa barplot command. For data analysis purposes, the frequencies of ASVs were collapsed at genus level using taxa *qiime taxa collapse* function. Relative abundance data at genus level was then used to test microbial difference between group using MaAsLin2 (67). Taxa present in at least 30% of the samples and those comprising more than 0.0001% of the total abundance were set as the cut-off criteria for further analysis. Post-FDR correction, a q-value of less than 0.05 was considered significant. Data can be visualized in **Table 1**.

Species richness was evaluated using α-diversity metrics, specifically the Shannon and Simpson indices, while differences in community composition (β-diversity) were analyzed using Bray-Curtis dissimilarity distances at the genus level. The Bray-Curtis dissimilarity measure utilized relative abundance data from each taxon across samples. Differences in β-diversity among groups were examined using PERMANOVA, with pairwise comparisons adjusted for multiple testing by the Benjamini-Hochberg method, employing the pairwiseAdonis R package (68–71). Plots were generated using ggplot2 with the *phyloseq* object.

### Immune cell isolation

Protocol was followed as previously published (45, 72, 73). In detail, small intestine, colon, pancreas, spleen were dissected and placed on ice-cold Dulbecco’s phosphate buffered saline (DPBS) (Brunschwig/Pan Biotech).

The mesenteric fat tissue and Peyer’s patches were removed from the intestine. The small intestine was cut longitudinally, washed of its contents and transferred into dissociation buffer (5 mM EDTA, 10 mM HEPES in 15 ml DPBS). Samples were incubated four times for 7 min at 37°C using a magnetic stirrer. Tissues were then shortly washed with IMDM and chopped before a performing a tissue digestion using a magnetic stirrer at 37°C for 20-30 min in digestion medium (0.5 mg/ml collagenase type VIII (Sigma), 10 U/ml DNase I (Roche) in 15 ml of IMDM), respectively. The obtained cell suspension was passed through a 100 µm cell strainer and washed in 20 ml of IMDM suppl. with 10% fetal calf serum (FCS). Cells were centrifuged at 2000 rpm for 7 min at 4°C and resuspended in 300 µl IMDM suppl. with 10% FCS.

Pancreatic lymph nodes were removed before mincing and digesting the pancreata using magnetic stirrer at 37°C for 15-20 min in digestion medium (0.5 mg/ml collagenase type IV (Worthington Biochemical), 10 U/ml DNase I (Roche) in 10 ml of IMDM suppl. with 2% FCS). The obained cell suspension was passed through a 100 μm cell strainer, washed with ice-cold phosphate buffered saline (PBS) and subsequently centrifuged at 2000 rpm for 7 min at 4°C. Then, they were incubated for 3 min in 1 ml of 1X red blood lysis buffer at room temperature (RT). 9 ml of ice-cold IMDM suppl. with 2% FCS was added to stop the reaction. Cells were recentrifuged and eventually resuspended in 200 µl of IMDM suppl. with 10% FCS.

Spleen was smashed through a 100 μm cell strainer using the plunger of a syringe. 5 ml of ice-cold IMDM suppl. with 10% FCS was added on top to rinse the cell strainer. Cells were centrifuged at 1500 rpm for 5 min at 4°C and resuspended in 1 ml of 1X of red blood lysis buffer (Invitrogen), and incubated for 2 min at RT. To stop the reaction, 9 ml of ice-cold IMDM suppl. with 10% FCS was added to stop the reaction. Cells were recentrifuged and resuspended in 1 ml of IMDM suppl. with 10% FCS.

### Flow cytometry

Single-cell suspensions were washed twice with 200 µl of IMDM suppl. with 10% FCS and incubated with a fixable viability dye (eFluor™ 506 or eFluor™ 450, Invitrogen) to exclude dead cells in DPBS (1:300). To avoid unspecific binding, a FC-Receptor blocking antibody against CD16/32 (Biolegend, clone 93) was added (1:400). Cells were stained for 20 min on ice in the dark and subsequently washed by adding 180 μl of FACS buffer (PBS, 2% FCS, 2 mM EDTA) and centrifuged at 2000 rpm for 2 min at 4°C. For the staining of surface markers, cells were resuspended in 50 μl of FACS buffer containing the appropriate amount of primary antibodies (**Table 4**) for 20 min on ice in the dark. Cells were washed and pelleted by centrifugation at 2000 rpm for 2 min at 4°C).

**Table 4:** Flow cytometry panel.

Antigen	Fluorochrome	Clone	Concentration	Company
Thy1	FITC	53-2.1	200	Thermofisher
CD19	APC	6D5	100	Biolegend
CD44	APC-Cyanine7	IM7	400	Biolegend
CD44	BV786	RM4-5	50	Biolegend
CD62L	BV510	MEL-14	100	Biolegend
TCRb	BUV395	H57-597	50	BD Biosciences
TCRgd	BUV737	Gl3	50	BD Biosciences
CD8	BV711	53-6.7	100	Biolegend
Foxp3	Alexa Fluor 700	FJK-16S	100	Thermofisher
Rorgt	BV650	Q31-378	1600	BD Biosciences
T-bet	PE-Cyanine7	4B10	1000	Thermofisher
Helios	PE-DAZZLE	22F6	200	Biolegend
Gata-3	PE	TWAJ	1000	Thermofisher

For intranuclear staining of transcription factors, cells were fixed and permeabilized with Foxp3/Transcription Factor Staining Buffer Set (Thermo Fisher Scientific) according to the manufacturer’s protocol for 45 min at 4°C in the dark. Afterwards, cells were washed twice with the provided buffer, and resuspended in 50 ul of provided buffer containing the appropriate concentration of antibodies (**Table 4**) and stained overnight in the dark at 4°C. The next morning, cells were washed with the provided buffer and resuspended in 200 µl of FACS buffer and measured using a LSRFortessa (BD Biosciences). Data were analyzed using FlowJoTM software version 10.9.0 (BD Biosciences).

In all experiments, FSC-H versus FSC-A was used to gate on singlets and a fluorescently-labeled fixable viability dye was used to remove dead cells. The gating strategy can be visualized in **Supplementary Figure 4**.

### ELISA

Testosterone concentrations were measured in the serum of mice using the Mouse Testosterone ELISA Kit (Crystalchem) according to the manufacturer’s instruction.

### Histology

Pancreata were fixed in 4% paraformaldehyde (PFA) for 4 h at 4°C and stored in 70% ethanol. The dehydration was done on the epredia CITADEL 2000 tissue processor, followed by paraffin embedding on the Epredia Shandon Histocentre 3.5 μm tissue sections were performed on the Microtome Leica RM 2155. Tissue sections were dewaxed in 100% xylol and decreasing concentrations of ethanol (100% - 50%).

### Hematoxylin and eosin staining

Hematoxylin staining was applied to tissue sections for 7 min, followed by thorough rinsing with water until clear. Subsequently, slides underwent a brief immersion in a solution of 0.5% hydrochloric acid-alcohol, followed by a more extended immersion in 70% ethanol. Eosin counterstaining was carried out for 3 min. The tissues underwent dehydration using progressively decreasing concentrations of ethanol and xylol before being mounted with entellan (Sigma-Aldrich). Slide scanning was performed at a 40X magnification using a 3DHistech Pannoramic 250 Flash II.

### Insulitis assessment

The insulitis severity was blindly assessed on six tissue sections per mouse. Scores for each islet (3-15 per section) were assigned based on criteria outlined in a previous study (74). The mean scores for each islet within the section was calculated, and then the average of all sections per mouse was determined. The scoring system is as follows: 0 indicates no insulitis, signifying the absence of infiltrates in the islet; 1 represents peri-vascular and peri-immune-islet infiltrates, where < 25% of the islet shows immune-islet infiltration, denoted as peri-insulitis; 2 signifies 25% to 75% of immune-islet infiltrates in the islets, indicating the presence of insulitis; and 3 indicates greater than 75% of immune-islet infiltrates in the islet, characterizing severe insulitis.

### Statistical analysis

If not stated otherwise, an unpaired parametric Student’s t-test was performed to compare two experimental groups in RStudio (Version 2023.06.0 + 421). If p-values are indicated with asterisks, the following applies *p < 0.05, **p ≤ 0.01, ***p ≤ 0.001, ****p ≤ 0.0001. Error bars indicate mean ± standard deviation.

## Data Availability

The 16S rRNA dataset supporting the conclusions of this article is available under https://doi.org/10.5281/zenodo.13771263. All other raw data supporting the conclusions of this article will be made available by the authors, without undue reservation.

## Supplementary material

Supplementary Figure 1 Colonization with the sDMDMm microbiota on the day of birth delays the development of type-1 diabetes. Type-1 diabetes incidence was assessed by biweekly monitoring of non-fasted blood glucose levels from 14 weeks onwards until disease onset or up to 1 year of age. Cumulative disease incidence in germ-free (GF) compared to GF animals colonized at birth with the sDMDMm microbiota (sDMDMm P0) in **(A)** female and **(B)** male NOD mice, respectively (n ≥ 26).

Supplementary Figure 2 Female NOD mice have an earlier type-1 diabetes development compared to male NOD mice. Type-1 diabetes incidence was assessed by biweekly monitoring of non-fasted blood glucose levels from 14 weeks onwards until disease onset or up to 1 year of age. Cumulative disease incidence in **(A)** germ-free (GF), **(B) **specific pathogen-free (SPF), GF mice colonized on the day of **(D)** birth (SPF P0), or** (E)** weaning (SPF P21), or **(E)** on the day of birth with the sDMDMm microbiota (sDMDMm P0) in NOD mice, respectively (n ≥ 26).

Supplementary Figure 3 Type-1 diabetes is delayed when mice are colonized at birth. Type-1 diabetes incidence was assessed by biweekly monitoring of non-fasted blood glucose levels from 14 weeks onwards until disease onset or up to 1 year of age. Cumulative disease incidence in GF animals colonized at birth (SPF P0) or weaning (SPF P21) with the SPF microbiota in **(A)** female and **(B)** male NOD mice, respectively (n ≥ 28).

Supplementary Figure 4 Gating strategy for T cell subsets. Single-cell suspensions were obtained from pancreas or small intestinal lamina propria (siLP) at postnatal day 56 or 21 and analyzed by flow cytometry. The gating strategy applied to a representative siLP sample to identify Th1 cells (T-bet^+^), Th2 cells (Gata-3^+^), Th17 cells (Rorgt^+^), and Treg subtypes (Rorgt^+^/ Helios^+^/Helios- Foxp3^+^).

Supplementary Figure 5 The presence of T helper cells during early life does not dictate microbiota-mediated protection against type-1 diabetes. Specific pathogen-free mice were injected with either an anti-CD4 or an isotype control antibody on postnatal days 10, 13, 16, 18, and 20. Type-1 diabetes incidence was assessed by monitoring non-fasted blood glucose levels, starting from 14 weeks and continuing until disease onset. Single-cell suspensions were obtained from small intestinal lamina propria (siLP) at postnatal day 20 (P20) and analyzed by flow cytometry. Cumulative disease incidence in **(A)** male and **(B) **female NOD mice, respectively (n ≥ 6 per group). **(C)** Gating strategy and indicated immune cell frequencies on siLP of control (Ctrl.) and CD4-depleted (CD4-depl.) NOD mice at P20 (n = 2). Data represent one experiment.
