# Supplementary figures and images for: The protective effect of the intestinal microbiota in type-1 diabetes in NOD mice is limited to a time window in early life
Source: Front Endocrinol (Lausanne). 2024 Sep 26;15:1425235. doi: 10.3389/fendo.2024.1425235 (PMC11464356; doi:10.3389/fendo.2024.1425235)

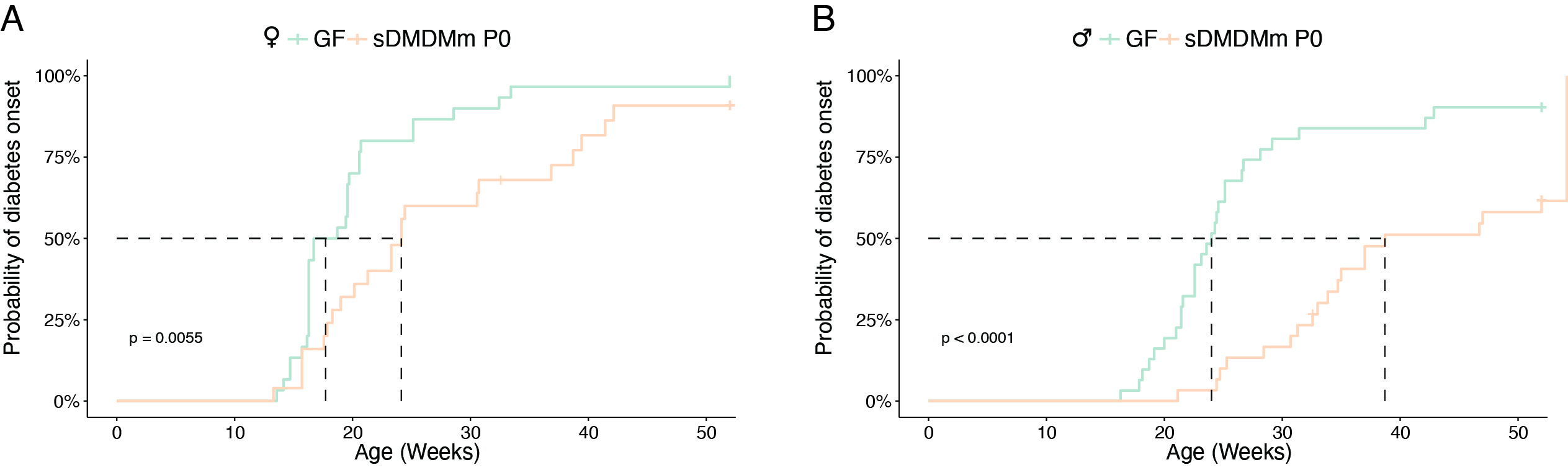

Supplement: Supplementary Figure 1 — Colonization with the sDMDMm microbiota on the day of birth delays the development of type-1 diabetes. Type-1 diabetes incidence was assessed by biweekly monitoring of non-fasted blood glucose levels from 14 weeks onwards until disease onset or up to 1 year of age. Cumulative disease incidence in germ-free (GF) compared to GF animals colonized at birth with the sDMDMm microbiota (sDMDMm P0) in (A) female and (B) male NOD mice, respectively (n ≥ 26). [file Image1.jpeg]

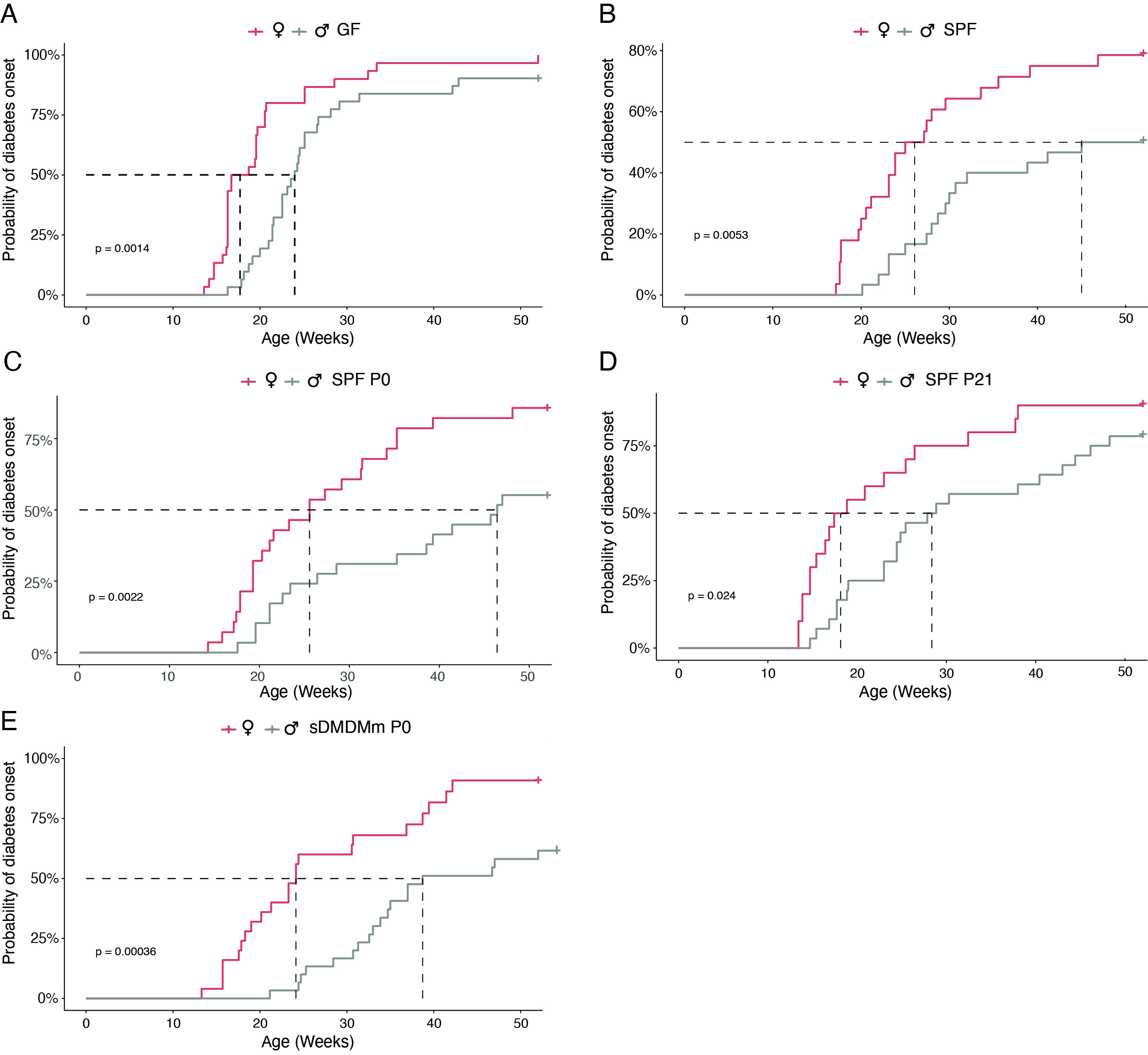

Supplement: Supplementary Figure 2 — Female NOD mice have an earlier type-1 diabetes development compared to male NOD mice. Type-1 diabetes incidence was assessed by biweekly monitoring of non-fasted blood glucose levels from 14 weeks onwards until disease onset or up to 1 year of age. Cumulative disease incidence in (A) germ-free (GF), (B) specific pathogen-free (SPF), GF mice colonized on the day of (D) birth (SPF P0), or (E) weaning (SPF P21), or (E) on the day of birth with the sDMDMm microbiota (sDMDMm P0) in NOD mice, respectively (n ≥ 26). [file Image2.jpeg]

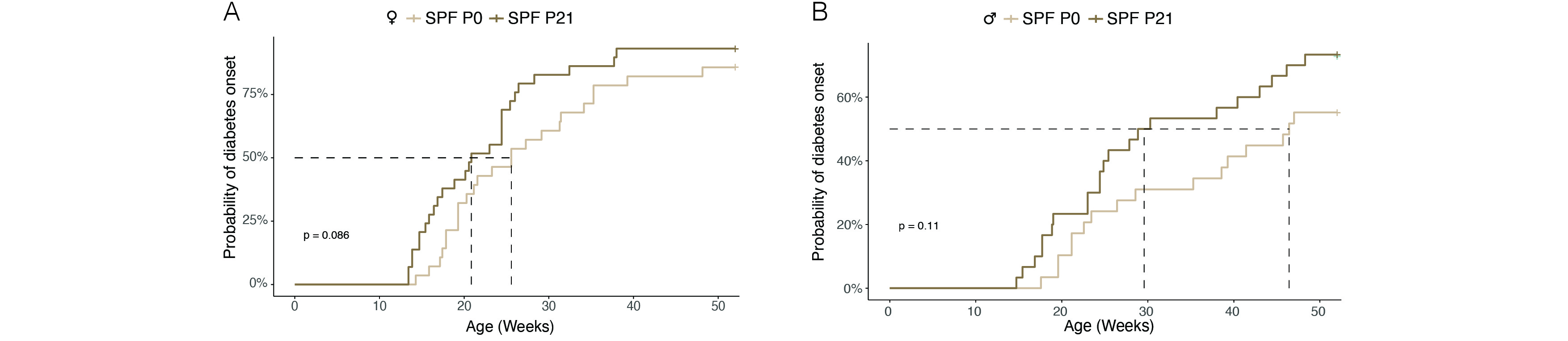

Supplement: Supplementary Figure 3 — Type-1 diabetes is delayed when mice are colonized at birth. Type-1 diabetes incidence was assessed by biweekly monitoring of non-fasted blood glucose levels from 14 weeks onwards until disease onset or up to 1 year of age. Cumulative disease incidence in GF animals colonized at birth (SPF P0) or weaning (SPF P21) with the SPF microbiota in (A) female and (B) male NOD mice, respectively (n ≥ 28). [file Image3.jpeg]

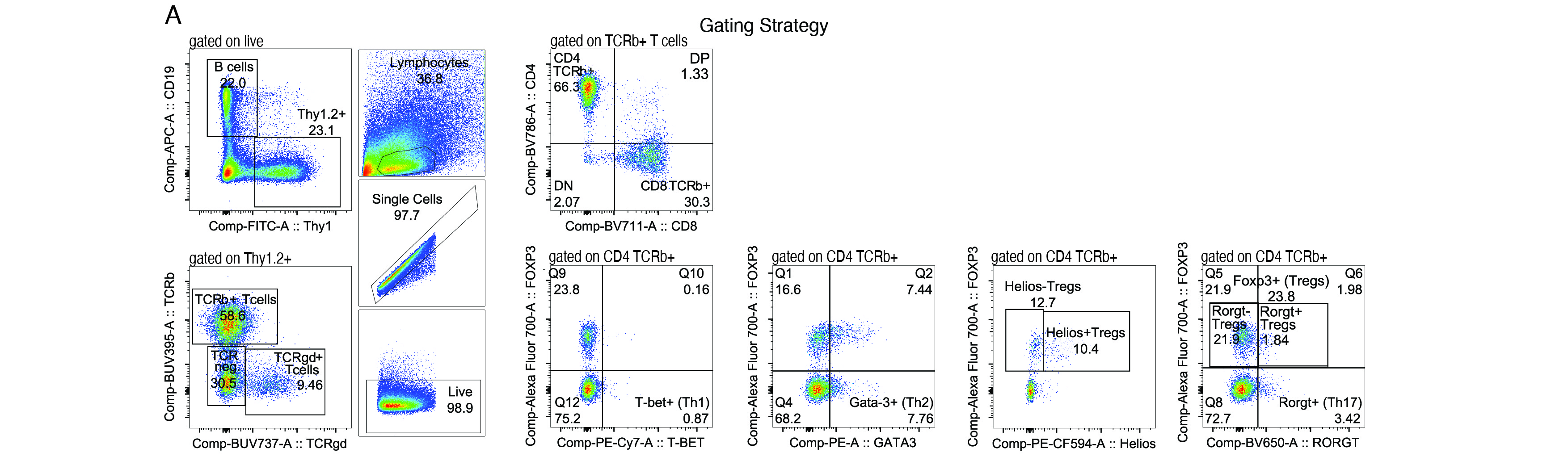

Supplement: Supplementary Figure 4 — Gating strategy for T cell subsets. Single-cell suspensions were obtained from pancreas or small intestinal lamina propria (siLP) at postnatal day 56 or 21 and analyzed by flow cytometry. The gating strategy applied to a representative siLP sample to identify Th1 cells (T-bet+), Th2 cells (Gata-3+), Th17 cells (Rorgt+), and Treg subtypes (Rorgt+/ Helios+/Helios- Foxp3+). [file Image4.jpeg]

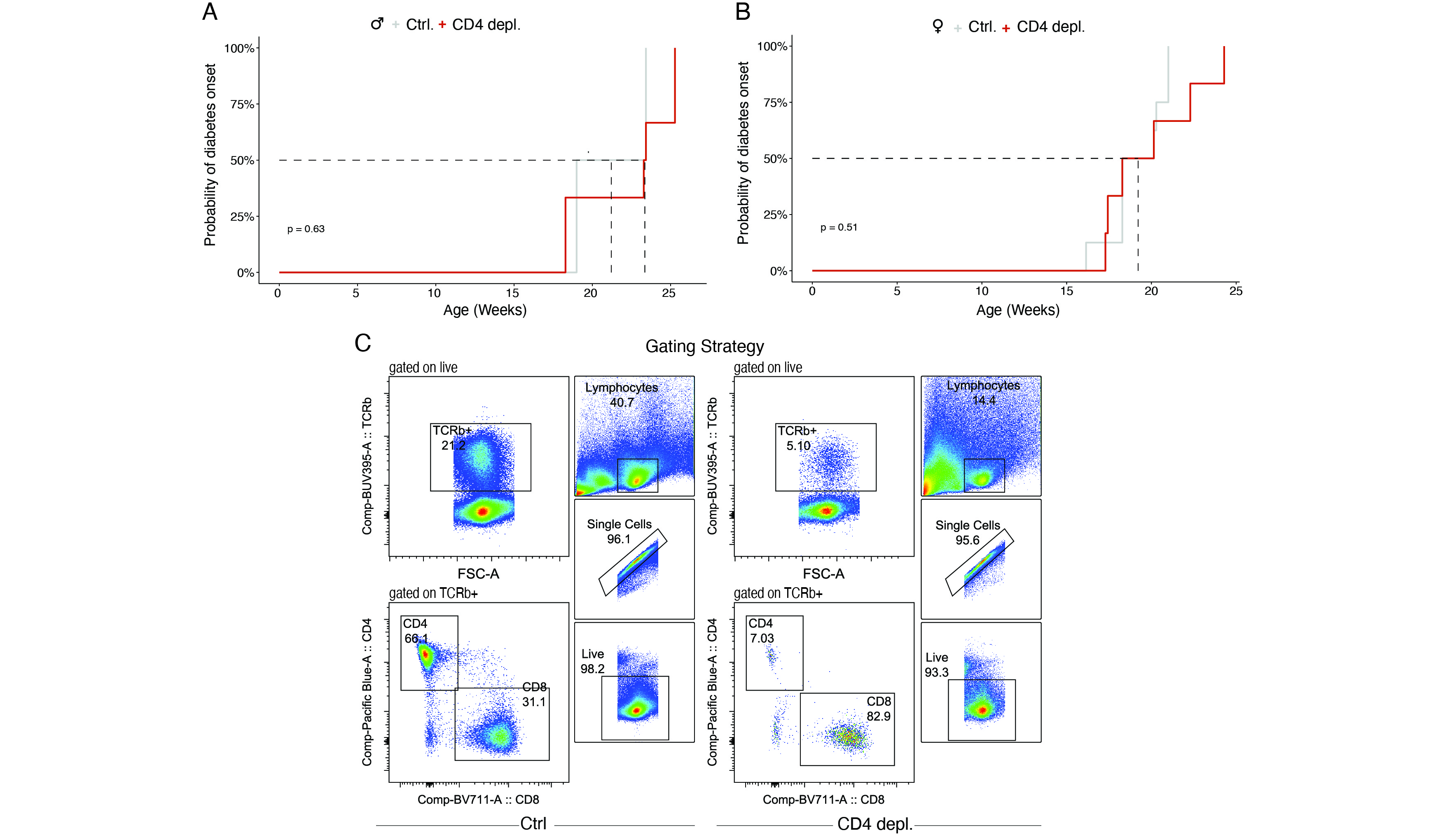

Supplement: Supplementary Figure 5 — The presence of T helper cells during early life does not dictate microbiota-mediated protection against type-1 diabetes. Specific pathogen-free mice were injected with either an anti-CD4 or an isotype control antibody on postnatal days 10, 13, 16, 18, and 20. Type-1 diabetes incidence was assessed by monitoring non-fasted blood glucose levels, starting from 14 weeks and continuing until disease onset. Single-cell suspensions were obtained from small intestinal lamina propria (siLP) at postnatal day 20 (P20) and analyzed by flow cytometry. Cumulative disease incidence in (A) male and (B) female NOD mice, respectively (n ≥ 6 per group). (C) Gating strategy and indicated immune cell frequencies on siLP of control (Ctrl.) and CD4-depleted (CD4-depl.) NOD mice at P20 (n = 2). Data represent one experiment. [file Image5.jpeg]
